# 347. Azole Activity against Filamentous Fungi Causing Invasive Infections in Patients from ICU and Non-ICU Units (2017-2021)

**DOI:** 10.1093/ofid/ofac492.425

**Published:** 2022-12-15

**Authors:** Cecilia G Carvalhaes, Paul Rhomberg, Paul Rhomberg, Beth A Schaefer, Michael A Pfaller, Mariana Castanheira

**Affiliations:** JMI Laboratories, North Liberty, Iowa; JMI Laboratories, North Liberty, Iowa; JMI Laboratories, North Liberty, Iowa; JMI Laboratories, North Liberty, Iowa; JMI Laboratories, North Liberty, Iowa; JMI Laboratories, North Liberty, Iowa

## Abstract

**Background:**

Invasive fungal infection (IFI) is associated with high mortality rates in critically ill patients. Appropriate antifungal treatment is crucial for managing IFI. We evaluated the *in vitro* activity of isavuconazole (ISC), itraconazole (ITC), posaconazole (PSC), and voriconazole (VRC) against contemporary moulds from ICU and non-ICU patients.

**Figure:**
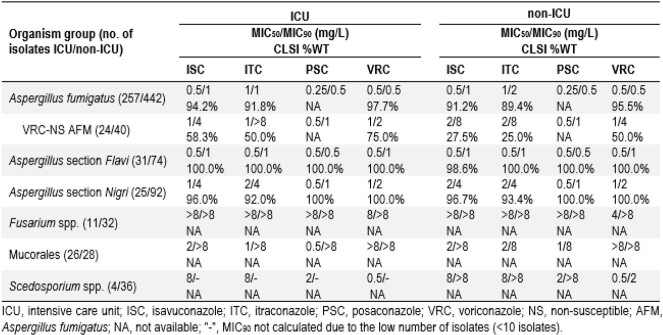

**Methods:**

1,226 moulds causing IFI (386/840 from ICU/non-ICU patients, respectively) were consecutively collected (1/patient) in 43 worldwide hospitals from 2017-2021 and susceptibility tested by CLSI broth microdilution. CLSI interpretation criteria were applied. Pneumonia was the predominant infection type among ICU (81.6%) and non-ICU (71.8%) patients.

**Results:**

*Aspergillus* spp. was the most common mould (87.6%/80.5%). *A. fumigatus* (AFM; 76.0%/65.4% of *Aspergillus*), *A*. section *Flavi* (ASF; 9.2%/10.9%), and *A.* section *Nigri* (ASN; 7.4%/13.6%) were the top 3 *Aspergillus* groups. ISC inhibited 94.2%/91.2%, 100.0%/98.6%, and 96.0%/96.7% of AFM, ASF, and ASN from ICU/non-ICU, respectively, at the epidemiological cut-off values. Similar activities were noted for ISC, PSC, ITC, and VRC against AFM from ICU (MIC_50/90_ range, 0.25-1/0.5-1 mg/L) and non-ICU (MIC_50/90_ range, 0.5-1/0.5-2 mg/L) isolates. No difference in the azole activities was noted against ASF from ICU (MIC_50/90_ range, 0.5/0.5-1 mg/L) and non-ICU (MIC_50/90_, 0.5/0.5-1 mg/L) or ASN from ICU (MIC_50/90_ range, 0.5-2/1-4 mg/L) and non-ICU (MIC_50/90_ range, 0.5-2/1-4 mg/L) isolates. VRC-non-susceptible (NS) AFM isolates were detected in 9.3% of ICU and 9.0% of non-ICU isolates. Among VRC-NS AFM from ICU/non-ICU, 58.3%/27.5% were wildtype (WT) to ISC, 50.0%/25.0% were ITC-WT, and 75.0%/50.0% remained VRC-WT, respectively. VRC was the most active azole against *Scedosporium* spp. (SCE) but was less active against Mucorales (MUC) than the other azoles. All azoles showed limited activity against *Fusarium* spp. (FUS). No difference on azole activities against SCE, MUC, and FUS were noted from ICU and non-ICU isolates.

**Conclusion:**

ISC was active against *Aspergillus* isolates from ICU and non-ICU, including against VRC-NS AFM from ICU. Azoles displayed similar activity against moulds from ICU and non-ICU units.

**Disclosures:**

**Cecilia G. Carvalhaes, MD, PhD**, AbbVie: Grant/Research Support|Cidara: Grant/Research Support|Melinta: Grant/Research Support|Pfizer: Grant/Research Support **Paul Rhomberg, BS, MT(ASCP)**, Cidara: Grant/Research Support|Pfizer: Grant/Research Support **Paul Rhomberg, BS, MT(ASCP)**, Cidara: Grant/Research Support|Pfizer: Grant/Research Support **Beth A. Schaefer, BA, MT(ASCP)**, Pfizer: Grant/Research Support **Michael A. Pfaller, MD**, Pfizer: Grant/Research Support **Mariana Castanheira, PhD**, AbbVie: Grant/Research Support|Cidara: Grant/Research Support|GSK: Grant/Research Support|Melinta: Grant/Research Support|Pfizer: Grant/Research Support|Shionogi: Grant/Research Support.

